# Monte Carlo Gradient Boosted Trees for Cancer Staging: A Machine Learning Approach

**DOI:** 10.3390/cancers17152452

**Published:** 2025-07-24

**Authors:** Audrey Eley, Thu Thu Hlaing, Daniel Breininger, Zarindokht Helforoush, Nezamoddin N. Kachouie

**Affiliations:** 1 Department of Mathematics and Systems Engineering, Florida Institute of Technology, Melbourne, FL 32901, USA; 2 Department of Electrical Engineering and Computer Science, Florida Institute of Technology, Melbourne, FL 32901, USA

**Keywords:** Gradient Boosted Trees, XGBoost, radiomics, lung cancer, imbalanced dataset, Monte Carlo

## Abstract

Machine learning algorithms are commonly employed for classification and interpretation of high-dimensional data. The classification task is often broken down into two separate procedures, and different methods are applied to achieve accurate results and produce interpretable outcomes. First, an effective subset of high-dimensional features must be extracted and then the selected subset will be used to train a classifier. Gradient Boosted Trees (GBT) is an ensemble model and, particularly due to their robustness, ability to model complex nonlinear interactions, and feature interpretability, they are well suited for complex applications. XGBoost (eXtreme Gradient Boosting) is a high-performance implementation of GBT that incorporates regularization, parallel computation, and efficient tree pruning that makes it a suitable efficient, interpretable, and scalable classifier with potential applications to medical data analysis. In this study, a Monte Carlo Gradient Boosted Trees (MCGBT) model is proposed for both feature reduction and classification. The proposed MCGBT method was applied to a lung cancer dataset for feature identification and classification. The dataset contains 107 radiomics which are quantitative imaging biomarkers extracted from CT scans. A reduced set of 12 radiomics were identified, and patients were classified into different cancer stages. Cancer staging accuracy of 90.3% across 100 independent runs was achieved which was on par with that obtained using the full set of 107 radiomics, enabling lean and deployable classifiers.

## Simple Summary

Lung cancer is the deadliest cancer worldwide, causing more annual deaths than breast, prostate, and colorectal cancer combined. In the U.S., it leads to approximately 340 deaths per day. Non-small-cell lung cancer (NSCLC) is the most common type, representing over 80% of cases. Due to its often-silent progression, lung cancer is commonly diagnosed at advanced stages, limiting treatment options and leading to poor survival rates. Only about 28% of patients survive beyond five years of diagnosis. Accurate staging, which assesses tumor size, lymph node involvement, and metastasis (TNM system), is critical for determining treatment and prognosis. Traditional imaging methods rely on visual interpretation, which can miss intangible features and vary between clinicians. To address these limitations, this study extracts imaging biomarkers called radiomics by quantitative feature extraction from CT scans. Advanced machine learning algorithms are implemented for analyzing radiomic patterns invisible to the human eye. The proposed model predicts lung cancer stages with high accuracy. The approach includes advanced techniques to manage data imbalance and reduce feature complexity without sacrificing performance, achieving over 90% accuracy. This methodology represents a significant step toward AI-driven precision oncology and is adaptable to other cancer types and imaging technologies.

## 1. Introduction

Lung cancer remains the leading cause of cancer-related mortality worldwide, accounting for more annual deaths than breast, prostate, and colorectal cancers combined [1]. According to the American Cancer Society, the disease causes approximately 340 deaths per day in the United States alone [1]. Lung cancer is broadly classified into two major types: non-small-cell lung cancer (NSCLC), which accounts for 80–85% of cases, and small-cell lung cancer (SCLC), a more aggressive variant responsible for 10–15% of cases [2,3]. The high lethality of lung cancer is due in part to its asymptomatic progression during early stages, resulting in late diagnoses when curative treatment options are limited [2]. Approximately 90% of lung cancer patients die within two years of diagnosis [4], underscoring the urgent need for better tools for early detection and accurate staging.

The staging of lung cancer is a critical clinical task that guides treatment decisions, determines prognosis, and shapes clinical trial eligibility. The TNM (tumor, node, metastasis) classification system developed by the American Joint Committee on Cancer (AJCC) and adopted globally by the Union for International Cancer Control (UICC) remains the standard framework for lung cancer staging [5]. This system evaluates (i) the size and extent of the primary tumor (T), (ii) the degree of regional lymph node involvement (N), and (iii) the presence of distant metastasis (M). Accurate staging is necessary not only for selecting appropriate treatment modalities, ranging from surgical resection to systemic therapies, but also for estimating survival and informing patient care pathways [5,6].

Traditional staging approaches rely heavily on anatomical imaging (e.g., CT, MRI, PET) interpreted qualitatively by radiologists. However, visual assessment is inherently subjective and can be prone to inter-observer variability. Furthermore, subtle or early-stage tumors may not present obvious radiographic signs, limiting detection accuracy. To overcome these limitations, a more objective, data-driven approach is essential, one that can extract and interpret patterns from medical imaging in a reproducible and scalable manner.

Radiomics is an emerging computational technique that enables the extraction of large numbers of quantitative imaging features from medical scans. These features capture information related to tumor shape, texture, intensity, and spatial relationships, many of which are invisible to the naked eye [7,8]. Radiomic analysis allows for a comprehensive characterization of tumor heterogeneity, which has been linked to treatment resistance and poor clinical outcomes [7,8]. Importantly, radiomics provides a non-invasive, whole-tumor assessment that avoids the sampling biases of traditional biopsies. For instance, in a seminal study by Coroller et al., 35 radiomic features were found to be predictive of distant metastasis in lung adenocarcinoma, with several features correlating strongly with patient survival [9].

To interpret the high-dimensional data produced by radiomic pipelines, machine learning algorithms are essential. Among them, Gradient Boosted Trees (GBT)**,** a type of ensemble model, is particularly well suited for medical applications due to its robustness, ability to model complex nonlinear interactions, and feature interpretability [10,11]. XGBoost (eXtreme Gradient Boosting) is a high-performance implementation of GBT that incorporates regularization, parallel computation, and efficient tree pruning, making it ideal for clinical research [12]. Previous work has demonstrated the power of XGBoost in a variety of biomedical tasks, including classification of chronic kidney disease [13], orthopedic outcome prediction [14], and breast and prostate cancer diagnostics [15,16]. In this study, we propose a reproducible and systematic machine learning pipeline that combines CT-derived radiomics with XGBoost for staging NSCLC. This work expands on our ongoing research into early cancer detection and classification using both imaging and genomic modalities [17,18,19,20]. Specifically, we execute the following:

- Introduce both binary and multiclass staging schemas to reflect real-world clinical stratification;
- Apply SMOTE to address class imbalance, comparing strategies applied before vs. after the train–test split;
- Evaluate performance across eight experimental dataflows incorporating full and reduced radiomic sets;
- Use feature importance analysis to identify a reduced subset of 12 biomarkers that maintain high classification accuracy.

Our approach conceptualizes radiomics as digital biosensors embedded in imaging data, capable of non-invasively sensing and reporting on tumor phenotype. We show that even a compact, carefully selected feature set can achieve results comparable to the full-feature set, with a maximum average accuracy of 90.3% over 100 model executions. This work lays a strong methodological foundation for intelligent imaging-based cancer staging. While we focus on lung cancer, the approach is broadly applicable to other malignancies and imaging modalities, supporting the future of precision oncology driven by artificial intelligence [21,22,23].

## 2. Data Description

To construct a robust machine learning framework for lung cancer staging, we utilized a publicly available dataset from the Cancer Imaging Archive (TCIA) curated by Aerts et al. [24]. This dataset comprises diagnostic imaging data and associated clinical information from patients diagnosed with non-small-cell lung cancer (NSCLC), the most common subtype of lung cancer [3]. Specifically, the cohort includes 398 patients for whom three-dimensional computed tomography (CT) scans and expert-annotated cancer staging labels are available. These patients span clinical stages I, II, IIIa, and IIIb, as defined by the American Joint Committee on Cancer TNM classification system [5,6].

### 2.1. Radiomic Feature Extraction

From each CT scan, 107 radiomic features were extracted using standardized radiomics pipelines. These features were derived from manually delineated tumor segmentations. Tumor annotations were generated by expert radiologists and reviewed for quality assurance. All features were extracted in three dimensions (3D) to fully capture volumetric tumor properties, and voxel intensity values were normalized to ensure inter-patient comparability. Feature extraction followed the definitions and formalism described by the PyRadiomics toolkit version 3 [25], a widely used open-source platform for radiomic analysis in medical imaging. Radiomic features were categorized into seven major classes, each representing a different aspect of tumor morphology or texture:

- First-order statistics: These features are computed from the distribution of voxel intensities within the region of interest (ROI), without accounting for spatial relationships. They include measures such as the mean, median, skewness, kurtosis, standard deviation, energy, entropy, and various percentile values. These features reflect overall intensity and heterogeneity and may correspond to variations in tumor density or internal necrosis [8];
- Shape features: These geometric descriptors quantify the 3D morphology of the tumor, independent of intensity. They include metrics such as volume, surface area, compactness, sphericity, elongation, and flatness. Shape features are especially valuable for staging, as higher-stage tumors often exhibit greater asymmetry and invasive spread into adjacent tissues [25];
- Texture features: These quantify the spatial relationships between voxel intensities and capture fine-grained heterogeneity patterns:
  ◦ GLCM (gray-level co-occurrence matrix) features describe the frequency of voxel pairs with specific intensity combinations, emphasizing local contrast and texture [8];
  ◦ GLSZM (gray-level size zone matrix) features capture the distribution of contiguous zones of uniform intensity, enabling assessment of tumor homogeneity or patchiness;
  ◦ GLRLM (gray-level run length matrix) features quantify the length of consecutive voxels with the same intensity along different directions, identifying directional textures or streaks;
  ◦ NGTDM (neighborhood gray tone difference matrix) features compute the contrast between a voxel intensity and the mean gray value of its neighbors, emphasizing local variation in gray tone [8];
  ◦ GLDM (gray-level dependence matrix) features assess the extent to which groups of voxels depend on a central voxel for their intensity, offering insights into complexity and coarseness [25].

### 2.2. Clinical Metadata and Feature Engineering

In addition to radiomic features, each patient record includes limited clinical data:

- Sex (male or female);
- Age (in years);
- Number of tumors, derived as a new feature from annotated lesion data.

Original entries corresponding to secondary lesions (i.e., metastatic foci within the lung) were aggregated into a single tumor count feature, representing the total number of tumors per patient. This transformation reduces dimensionality and avoids artificially inflating the sample size due to repeated measures from the same individual.

### 2.3. Data Cleaning and Filtering

To ensure analytical integrity, the following preprocessing steps were applied:

- Records with missing values in radiomic or clinical fields were excluded;
- All features were standardized using z-score normalization to zero-mean and unit-variance across the dataset;
- The final analytic dataset included 398 complete cases, with balanced representation across sex and a wide age distribution.

### 2.4. Cancer Staging Labels

Ground-truth cancer stage labels were derived from clinical records and categorized based on the TNM staging system [5,6]:

- Stage I: Localized tumor without lymph node involvement or metastasis;
- Stage II: Larger primary tumor or minor nodal involvement;
- Stage IIIa: Tumor spread to mediastinal lymph nodes on the same side of the chest [26];
- Stage IIIb: Contralateral or subcarinal lymph node involvement, often precluding surgical resection [27].

For modeling purposes, these stages were used to generate the following:

- Binary class labels (early vs. advanced stage);
- Three-class labels (low, medium, high severity) to better reflect clinical stratification.

The availability of radiomic, clinical, and staging data in a unified dataset makes this cohort ideal for machine learning research in lung cancer staging.

## 3. Methods

**Figure 1:**
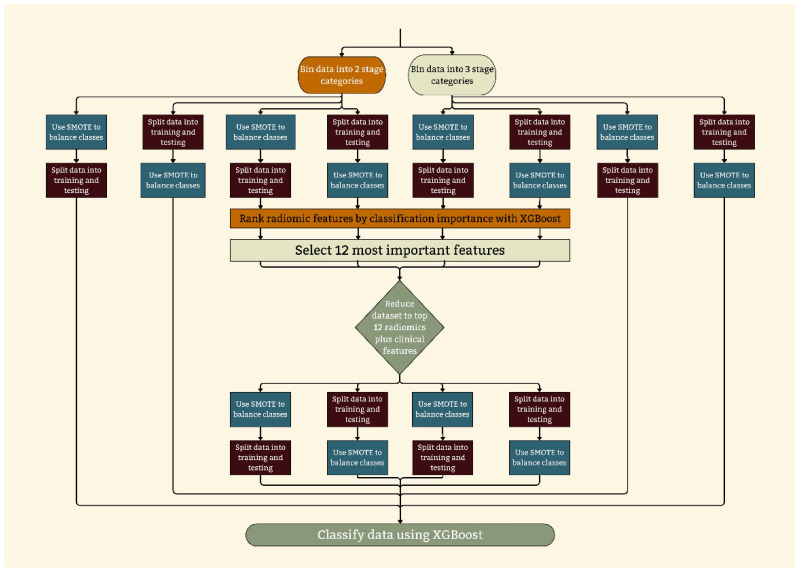
Workflow diagram of the proposed method. Eight combinations of the feature set, oversampling strategy, and stage classification labeling were created.

The proposed methodological strategy for the stratification and predictive classification is discussed in this section. Depicted in Figure 1, eight data pipelines were created by a full and reduced radiomics feature set, two binning techniques, and two different oversampling strategies.

### 3.1. Binary and Multiclass Binning

The problem of cancer staging from imaging biomarkers can be approached as a classification task, where patients are assigned to discrete categories based on their tumor characteristics. However, lung cancer staging is inherently ordinal, meaning that the classes (e.g., Stage I through IV) have a natural progression in terms of severity and prognosis. Designing an effective machine learning model thus requires careful consideration of how these stages are grouped and labeled. In this study, two parallel classification schemas were implemented: a binary classification and a multiclass classification. These schemas were designed not only to test algorithmic robustness under varying complexity but also to simulate two common use-cases in clinical practice: (i) early detection (binary), and (ii) fine-grained stratification for treatment planning (multiclass).

#### 3.1.1. Binary Classification: Early vs. Advanced Stages

For the binary classification task, we grouped patients into two classes:

- Low stage: includes patients diagnosed with Stage I and Stage II;
- High stage: includes patients with Stage IIIa and IIIb.

This binning reflects a clinically meaningful distinction between tumors that are generally resectable (Stages I–II) and those that typically require non-surgical, multimodal therapy (Stages IIIa–IIIb) due to local advancement or nodal involvement. The rationale behind combining I and II into a single class lies in their relatively favorable prognosis and eligibility for curative interventions like lobectomy or stereotactic radiotherapy [5]. From a machine learning perspective, binary classification allows for the following:

- Simpler decision boundaries in feature space;
- More stable learning under class imbalance;
- Clearer evaluation metrics such as sensitivity and specificity.

Binary staging classification also aligns with real-world screening scenarios, where the primary question is whether the detected tumor suggests early- or late-stage disease.

#### 3.1.2. Multiclass Classification: Three-Stage Stratification

In the multiclass procedure, patients were assigned to three ordinal classes according to clinical stage gradation:

- Stage I (low): localized tumors with no lymphatic spread;
- Stage II (medium): tumors with greater size or minor lymph node involvement;
- Stages IIIa and IIIb (high): regionally advanced tumors with significant nodal involvement.

While Stages IIIa and IIIb have different anatomical criteria [26,27], they were merged due to limited sample sizes and similar treatment implications (e.g., induction chemotherapy, unrespectability). This decision also ensured that class frequencies remained reasonably balanced. Multiclass classification is a more challenging task, particularly in high-dimensional spaces, due to the following:

- Increased overlap between classes in feature space;
- Greater susceptibility to data imbalance;
- Need for multiclass-capable classifiers (e.g., one-vs.-rest or SoftMax-based models).

Nonetheless, the multiclass setting provides greater granularity for clinical interpretation, enabling stage-specific recommendations and more nuanced risk stratification.

#### 3.1.3. Label Encoding for Modeling

Class labels were encoded numerically:

- For binary classification:
  ◦ 0: early stage (Stage I and II);
  ◦ 1: late stage (Stage IIIa and IIIb).
- For multiclass classification:
  ◦ 0: Stage I;
  ◦ 1: Stage II;
  ◦ 2: Stage IIIa or IIIb.

This label encoding was compatible with the input expectations of the XGBoost classifier, which supports both binary and multiclass modes through parameter flags (objective: “binary:logistic” or “multi:softmax”). Both classification schemas were evaluated independently using the same feature sets and model configurations to allow direct performance comparisons across binary and multiclass tasks.

### 3.2. Addressing Class Imbalance

After data stratification was performed, severe class imbalances were apparent regardless of the binning strategy. It was crucial to address the imbalance issue as it has the adverse effect on the training of the classifier.

#### 3.2.1. Nature of the Imbalance

One of the fundamental challenges in clinical machine learning, particularly in cancer datasets, is class imbalance. Real-world medical datasets are rarely balanced across disease stages, since some stages are more prevalent in the population than others. In the dataset in this study with 398 NSCLC patients, this imbalance was significant:

- Stage IIIa and IIIb (advanced stages) accounted for 277 patients (~70%);
- Stages I and II (early stages) were underrepresented, with only 121 patients (~30%).

This imbalance was even more pronounced in the multiclass setup. For example, Stage II had far fewer patients than Stage I or III, potentially skewing the model toward over-predicting the majority class (Stage III). Without correction, such imbalance would cause the model to prioritize majority-class accuracy, leading to poor sensitivity (true positive rate) for minority classes, a critical flaw in clinical decision support.

#### 3.2.2. SMOTE: Synthetic Minority Oversampling Technique

To mitigate class imbalance, the Synthetic Minority Oversampling Technique (SMOTE) was implemented [28]. SMOTE is an algorithm that generates synthetic examples for the minority class by interpolating between neighboring feature vectors. Specifically, it performs the following:

- For each instance in the minority class, a number of K-nearest neighbors (typically K = 5) are identified;
- New instances are created by taking the vector difference between a sample and its neighbors, multiplying by a random scalar between 0 and 1, and adding it to the original vector;
- This results in synthetic examples that lie within the convex hull of existing data points.

Unlike simple duplication, SMOTE avoids overfitting by introducing variability while preserving class boundaries. It is particularly well suited for high-dimensional data such as radiomics, where feature-space sparsity can exacerbate minority class exclusion during training [10].

#### 3.2.3. Oversampling Strategies

To explore how the order of oversampling affects model performance, we implemented and compared two SMOTE strategies:

Balanced–Balanced (B) Technique: SMOTE Before Train–Test Split

In this approach, SMOTE was applied to the entire dataset before partitioning into training and test sets:

- All class labels were balanced to contain 277 instances each;
- The balanced dataset was then split into 80% training and 20% testing.

Advantages:

- Training and test sets are drawn from an already-balanced distribution;
- The classifier is evaluated on samples from the same (balanced) feature space.

Risks:

- Synthetic examples may appear in both training and test sets, introducing data leakage;
- Overestimates generalization if the test set is not representative of real-world distributions.

In summary, synthetic records, while potentially not an exact match to real patient data for every cancer stage, are generated with values that fall within the permissible range for each biomarker and are based on the characteristics of nearby data points. This process facilitates improved learning by introducing richer data variations and including potential patient profiles not captured in the original dataset. Consequently, the synthesized data enhance model diversity and mitigate overfitting.

Balanced–Original (O) Technique: SMOTE After Train–Test Split

In this more conservative strategy, SMOTE was applied only to the training set after splitting:

- The training set was oversampled to balance class distributions;
- The test set retained its original imbalanced distribution.

Advantages:

- Preserves the natural distribution in the test set;
- Offers a more realistic estimate of model performance in the real world.

Challenges:

- Training is more difficult due to fewer original examples in minority classes;
- May underperform in accuracy, but with less risk of overfitting.

#### 3.2.4. Integration into the Pipeline

Both SMOTE strategies were integrated into the modeling pipeline across binary and multiclass tasks. This yielded four oversampling configurations:

- Binary—Strategy B (SMOTE before): denoted as FDBB and RDBB;
- Binary—Strategy O (SMOTE after): denoted as FDBO and RDBO;
- Multiclass—Strategy B: FDMB and RDMB;
- Multiclass—Strategy O: FDMO and RDMO.

These configurations allowed systematic evaluation of the interaction between the following:

- Oversampling timing;
- Classification granularity;
- Feature-set size (full vs. reduced).

In total, eight dataflows were analyzed (discussed in Section 3.4), enabling a comprehensive sensitivity analysis.

### 3.3. Gradient Boosted Trees (GBT)

XGBoost (eXtreme Gradient Boosting) was implemented for both feature ranking and cancer staging. The use of machine learning algorithms in medical diagnostics necessitates a careful balance between **predictive performance**, **interpretability**, and **computational efficiency** where XGBoost as a state-of-the-art and scalable implementation of Gradient Boosted Trees (GBT) has gained widespread adoption in structured-data problems, particularly in clinical applications [10,12]. GBT algorithms are a class of **ensemble learning methods** that combine the predictive power of many weak learners (typically decision trees) to form a strong learner. Each new tree is trained to correct the residual errors of the previous ensemble, gradually improving overall prediction accuracy. Unlike Random Forests, which build trees independently and average their outputs, GBT builds trees sequentially, with each new tree focusing on minimizing the loss function of the ensemble thus far [11].

#### 3.3.1. Mathematical Formulation

Given a dataset $D={\left\{\left(x_{i},\;y_{i}\right)\right\}}_{i=1}^{n}$ where each $x_{i}\in R^{d}$ is a feature vector of radiomics and clinical variables and $y_{i}$ is the class label (cancer stage) where $y_{i}\in \left\{0,\;1\right\}$ for binary and $y_{i}\in \left\{0,\;1,\;2\right\}$ for multiclass classification, the prediction model is as follows:

(1) $${\hat{y}}_{i}=\sum_{k=1}^{K}f_{k}\left(x_{i}\right)$$

where each $f_{k}$ is a regression tree and *K* is the total number of trees. The model optimizes an objective function composed of a regularized loss:

(2) $$L\left(\phi \right)=\sum_{i=1}^{n}l\left(y_{i},\hat{y_{i}}\right)+\sum_{k=1}^{K}\Omega \left(f_{k}\right)$$

where *l* is a differentiable loss function, i.e., logistic loss for binary and softmax for multiclass classification, and the regularization term $\Omega \left(f\right)$ is as follows:

(3) $$\Omega \left(f\right)=\gamma T+\frac{1}{2}\lambda {\left|\left|w\right|\right|}^{2}$$

To penalize complexity, *T* is the number of leaf nodes, and *w* is magnitude of leaf weights. Gradient boosting proceeds in an additive fashion by fitting each tree $f_{k}$ to the negative gradient of the loss with respect to the current model’s predictions. This approach enables fast convergence and robust error correction over iterations.

#### 3.3.2. Feature Handling

XGBoost handles missing values internally by learning optimal splitting directions when a missing feature is encountered. However, in our case, all incomplete entries were excluded during preprocessing (Section 2.3) to simplify reproducibility.

All features were standardized using z-score normalization, which is not required for tree-based models but aids in interpretability when visualizing feature distributions and selecting thresholds (e.g., in violin plots or decision paths).

The model was trained on two distinct feature sets:

- Full-feature set: all 107 radiomic features + age, sex, and tumor count;
- Reduced-feature set: the top 12 radiomic features (Section 3.4) + same clinical variables.

The integration of both clinical and radiomic data allowed the model to exploit imaging-derived biomarkers alongside simple demographic features, mimicking real-world electronic health records.

#### 3.3.3. XGBoost for Radiomic Feature Identification

Radiomic features are high-dimensional, sparse, and often collinear conditions under which traditional statistical models (e.g., logistic regression) underperform due to multicollinearity and limited expressiveness. Deep learning methods, while powerful, require much larger datasets and are less interpretable. XGBoost, in contrast, achieves the following:

- Automatically handles feature interaction via hierarchical tree splits;
- Provides feature importance scores, enabling selection and interpretation;
- It is robust to outliers;
- Trains efficiently on small to moderate datasets, which is typical in oncology.

Its performance, stability, and explainability make XGBoost particularly well suited for use in clinical radiomics-based decision support systems [12,13,14,15,29,30,31].

### 3.4. Classification Strategies and Feature Selection

The data pipelines (Figure 1), feature reduction, and validation procedures are explained in this section.

#### 3.4.1. Dataflow Naming and Strategy Design

To systematically evaluate how different methodological decisions impact model performance, a total of eight distinct classification pipelines were constructed. Each pipeline represents a unique combination of three key methodological axes:

- Feature set: full (F) vs. reduced (R);
- Classification type: binary (B) vs. multiclass (M);
- Oversampling strategy: SMOTE before (B) vs. SMOTE after (O) train–test split.

Each pipeline was encoded using a four-letter abbreviation, where each letter corresponds to one of the conventions below:

- First letter: F = full dataset, R = reduced dataset;
- Second letter: D = dataset context;
- Third letter: B = binary classification, M = multiclass classification;
- Fourth letter: B = SMOTE before split, O = SMOTE after split.

The pipeline definitions are shown in Table 1.

Each pipeline was executed 100 times to reduce the influence of stochastic variability (e.g., random splits, SMOTE interpolation, XGBoost initialization). For each execution, the following was performed:

- The dataset was partitioned into 80% training/20% testing;
- SMOTE was applied according to the strategy;
- XGBoost was trained using standardized default hyperparameters (Appendix A.1);
- Predictions on the test set were recorded;
- Accuracy, sensitivity, specificity, F1 score, and confusion matrices were computed.

Averaging these metrics across runs provided stable performance estimates for all pipelines. This exhaustive design enables comparative analysis of the following:

- Feature reduction effects (full vs. reduced);
- Class structure impact (binary vs. multiclass);
- Oversampling effects on generalization (Strategy B vs. O).

Figure 1 illustrates the data flow of each of these pipelines. From left to right, the pipelines are as follows: FDBB, FDBO, RDBB, RDBO, RDMB, RDMO, FDMB, and FDMO.

#### 3.4.2. Feature Importance-Based Reduction

Listed in Table A1 (in Appendix A.2), the full-feature set contains 107 radiomic features using the full set of radiomics for classification is challenging due to the following:

- High dimensionality: increases the risk of overfitting;
- Redundancy: many features are redundant or weakly informative;
- Computational cost: scales with feature count.

To address these issues, a reduced-feature set (RFS) was constructed by identification of impactful features using importance scores.

Importance Scoring Procedure

During model training, XGBoost computes importance scores for each feature based on the following:

- Gain: The average reduction in loss (e.g., log-loss) when a feature is used for splitting;
- Weight: The number of times a feature is used in all splits;
- Cover: The number of samples affected by the splits using this feature.

The gain metric, which emphasizes predictive contribution over frequency, was employed. A stable ranking was obtained by the following:

1. A Monte Carlo approach was used to assess the impact of randomness by running the FDBB model (full, binary, SMOTE before) multiple times (100 times in this study);
2. In each run, the gain-based importance of each feature was recorded;
3. Importance scores for each feature were averaged across multiple runs;
4. Features were ranked by their mean importance.

Top 12 Features Selected

**Figure 2:**
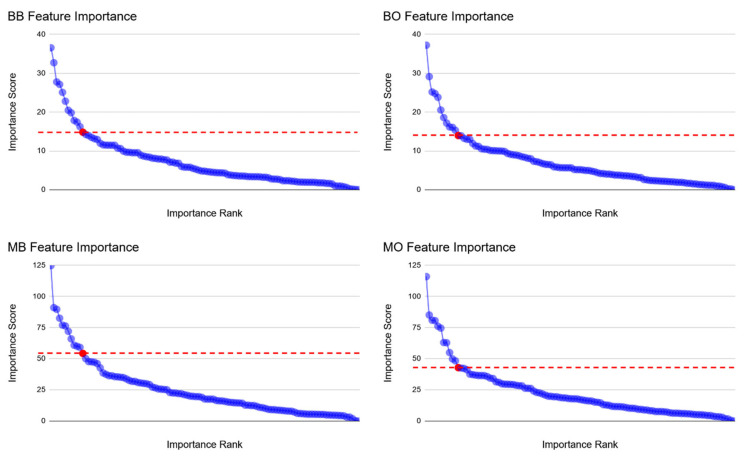
The distribution of importance scores for all 107 radiomic features, plotted in descending order, with the red data point marking the 12th most important feature (red dashed line demonstrate the cutoff for the reduced dataset). Binary (top row) vs. multiclass (bottom row) classification; SMOTE before (left column) vs. SMOTE after (right column).

The reduced-feature set identified by each data pipeline contains the top 12 radiomics, ranked as the most important features. The top 12 radiomic features were selected based on a noticeable drop-off in the importance score after the 12th rank (Figure 2). The union of four reduced-feature sets contains 18 radiomics that are listed in Table 2.

#### 3.4.3. Validation of Feature Reduction

To validate that performance was retained in the reduced-feature models, we re-ran all pipelines (RDBB, RDBO, RDMB, RDMO) using only the top 12 features and the 3 clinical features. The results were compared to their full-feature counterparts (FDBB, etc.) and showed only marginal reductions in accuracy, often less than 1%. This supports the hypothesis that a small number of well-selected radiomics can serve as a compact biosensor signature for tumor staging. Moreover, reduction in dimensionality enabled the following:

- Faster training and evaluation;
- Improved model interpretability;
- Potential for hardware-efficient deployment in clinical environments.

Figure 2 presents the radiomics importance scores, organized by the four different feature reduction pipelines used. In every panel, one particular feature stands out as significantly more important than the others. Looking specifically at the binary classification panels (top row), the importance scores of the features that follow the most dominant radiomic are clustered more closely together contained within a smaller range in comparison with the multiclass scores (bottom row). For pipelines where the test data were not oversampled (right column), the most important radiomics formed noticeable clusters with comparable importance scores. In contrast, pipelines that utilized oversampled test data (left column) displayed a pattern where the importance scores of the succeeding radiomics were spaced apart after the most important feature. A consistent observation across all panels is a decreasing exponential trend of importance scores after the red-indicated cutoff point.

## 4. Results

This section presents the analytical outcomes of our methodological study, emphasizing how different classification strategies, sampling protocols, and feature selection approaches affect model performance. By dissecting results across multiple axes, feature relevance, inter-feature relationships, classifier decision logic, and prediction accuracy, we provide a robust validation of our proposed machine learning pipeline for lung cancer staging. Monte Carlo results are derived from 100 repeated model executions per configuration, ensuring statistical stability and minimizing randomness due to train–test splits or oversampling noise.

### 4.1. Feature Importance and Interpretability

The XGBoost model architecture naturally provides feature importance scores, enabling interpretability by quantifying each feature’s contribution to decision-making. As described in Section 3.4, the average Monte Carlo importance scores were calculated across 100 executions of the FDBB pipeline to create a stable, reproducible ranking. Figure 2 shows this distribution, where the importance of all 107 radiomic features is plotted in descending order. Several key insights emerged:

- Sphericity consistently ranked number one, suggesting that tumors with more irregular or elongated shapes (lower sphericity) were strongly associated with advanced stages. These matches established clinical observations that advanced-stage lung tumors are more invasive and spatially heterogeneous [8,9];
- Elongation and flatness, both geometric descriptors, also ranked in the top five, indicating that shape complexity is a dominant signal in staging classification;
- First-order features such as skewness, median, and maximum 3D diameter ranked high as well. These features likely capture aspects of tumor density, asymmetry in intensity distribution (e.g., due to necrosis), and size, all known correlates of disease progression;
- Texture features (e.g., GLDM dependence entropy, GLCM contrast) appeared lower in the ranking but still contributed to performance. Their value may be more nuanced, distinguishing between medium and high stages, or capturing subtle heterogeneity not seen in geometry alone.

The 12th feature in the ranking showed a marked drop in importance, providing a natural cutoff for constructing a reduced-feature set. This threshold was consistent across repeated runs and model types.

**Figure 3:**
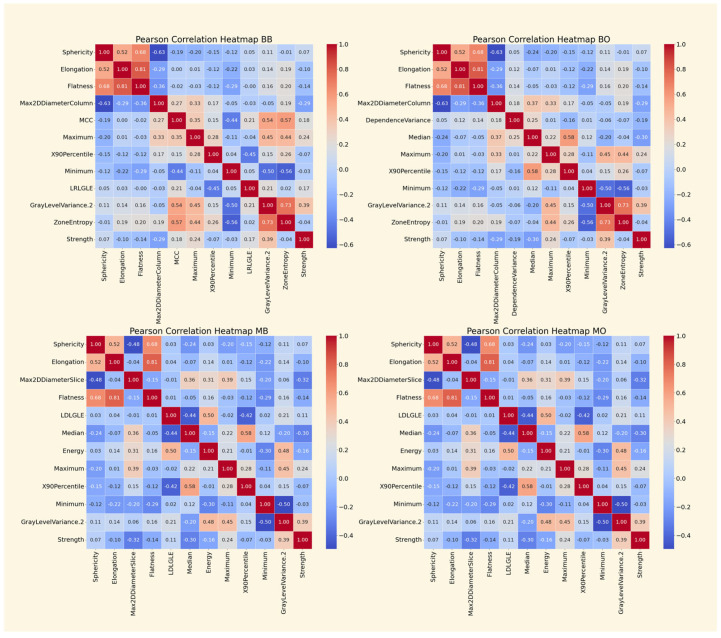
Pearson correlation matrices of top 12 radiomics for each reduced-feature set. Binary (top row) vs. multiclass (bottom row) classification; SMOTE before (left column) vs. SMOTE after (right column).

The heatmaps featured in Figure 3 visualize the Pearson correlation coefficients of the radiomic features in each of the reduced radiomic sets. Across all panels, sphericity, elongation, and flatness are included in the reduced set and are correlated to some degree. The correlation of flatness and elongation is especially strong (0.81). Given that these features relate to tumor shape, it is somewhat intuitive that such correlations exist. Notably, features describing the maximum diameter of the tumor are negatively correlated with sphericity. This indicates that tumors that are more spherical have smaller diameters than those that are less so. In the binary correlation matrices (top row), zone entropy and gray-level variance 2 are shown to be highly correlated (0.73). These features describe different aspects of texture, with higher values for both metrics indicating highly variable tumor texture. Although the features were not ranked in the same order, the reduced multiclass feature sets (bottom row of panels) contain all of the same radiomics.

### 4.2. Redundancy and Complementarity in Radiomic Features

To ensure that the top-ranked features used in reduced models were not redundant, we examined Pearson correlation matrices for the reduced-feature sets (Figure 3).

The results revealed the following:

- High correlation among shape features, particularly between sphericity, elongation, and flatness. While this may suggest overlap, each metric captures a different aspect of morphology (e.g., sphericity measures roundness, elongation measures axis distortion), justifying their inclusion;
- First-order features such as median and skewness showed moderate to low correlation, indicating they offer orthogonal information about tumor intensity distribution;
- Texture features were weakly correlated with both shape and statistical features, confirming their complementary value. For instance, GLDM dependence entropy was uncorrelated with geometric features but contributed to distinguishing among mid-stage cases (e.g., Stage II).

These findings validate that our reduced-feature set is informative, diverse, and contains non-redundant critical qualities for robust model generalization and interpretability.

### 4.3. Decision Tree Interpretability

**Figure 4:**
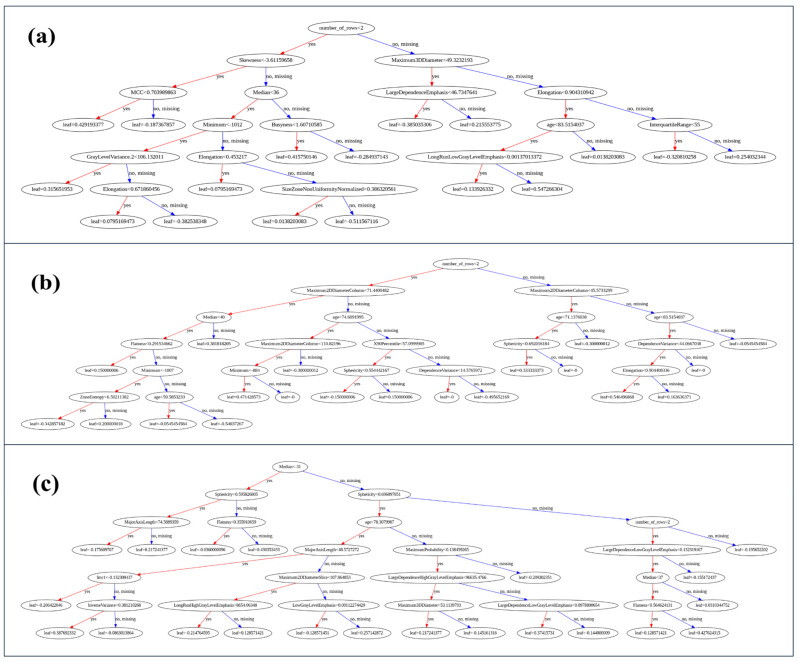
(**a**) XGBoost tree produced from an execution of the FDBB model. (**b**) XGBoost tree produced from an execution of the RDBO model. (**c**) XGBoost tree produced from an execution of the FDMO model.

Figure 4 provides a snapshot of representative XGBoost trees obtained through different data flow pipelines. Panel (a) illustrates how the model incorporates feature logic into stage predictions:

- The root node splits on “Number of Rows”, a clinical feature derived from patient metadata, and represents the number of tumors a patient has. This reflects the fact that patients with multiple nodules are more likely to be at advanced stages due to intrapulmonary metastasis or synchronous tumors;
- Subsequent splits use skewness, maximum 3D diameter, and sphericity, highlighting a hierarchy where the model first evaluates tumor burden and size, then examines shape irregularity;
- Leaf nodes assign probabilities to stage labels, allowing soft decision-making.

The tree in panel (b) has some similarities to panel (a) but has key differences that demonstrate the random nature of XGBoost, including the following:

- The number of tumors is the root node similar to panel (a);
- The first split is based on the shape feature, “maximum 2D diameter column”;
- Then, “age” is a major consideration, along with “zone entropy” and “dependence variance”;
- The remaining splits are based on shape features and first-order statistics.

Panel (c) incorporates more nodes than the previous decision trees in (a) and (b). However, the splits are mainly based on similar features:

- The first split is based on the “median”, a first-order statistics feature, that corresponds to the median density of cancerous tissue. It was identified as the top 12 impactful radiomics by the proposed feature selection procedure;
- The next set of splits is entirely based on the value of the “sphericity”, a shape feature;
- The next set of node splits is based on the shape category of radiomics along with the clinical features;

The remaining decision nodes on the right side of the tree are mainly based on radiomics extracted from the CT scan gray-level intensities along with shape features. This interpretability is critical in clinical contexts, where model output must be trace-able and justifiable. Unlike black-box neural networks, XGBoost trees provide a transparent decision structure compatible with regulatory standards and physician expectation.

### 4.4. Classification Accuracy and Comparative Pipeline Performance

To evaluate the impact of methodological design choices, the performance across all eight pipeline configurations was compared by the following:

- Overall accuracy;
- Class-specific metrics (TPR, FPR, TNR, FNR);
- Effect of order when balancing the dataset (using SMOTE);
- Full- vs. reduced-feature set.

#### 4.4.1. Binary vs. Multiclass Confusion Matrices

**Figure 5:**
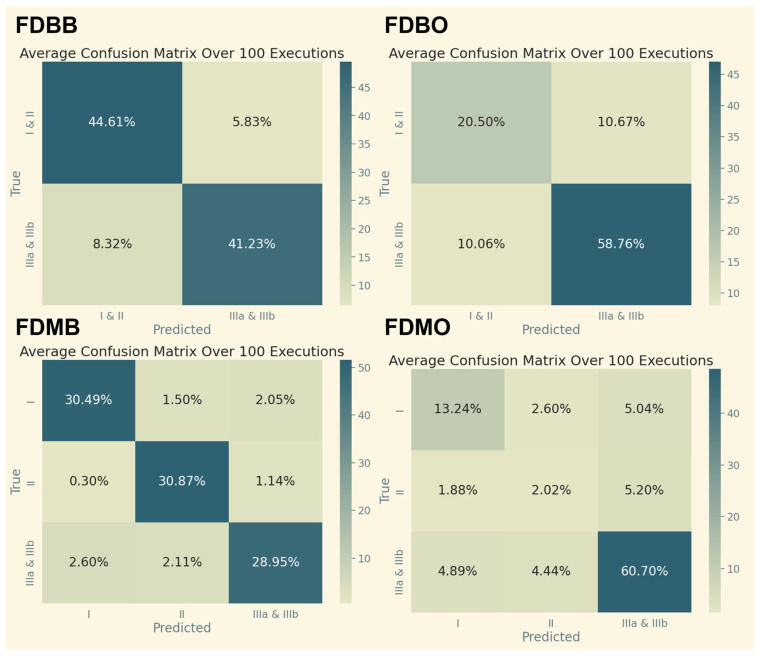
Confusion matrices from XGBoost classifications using the full dataset averaged over 100 executions. Binary (top row) vs. multiclass (bottom row) classification; SMOTE before (left column) vs. SMOTE after (right column).

**Figure 6:**
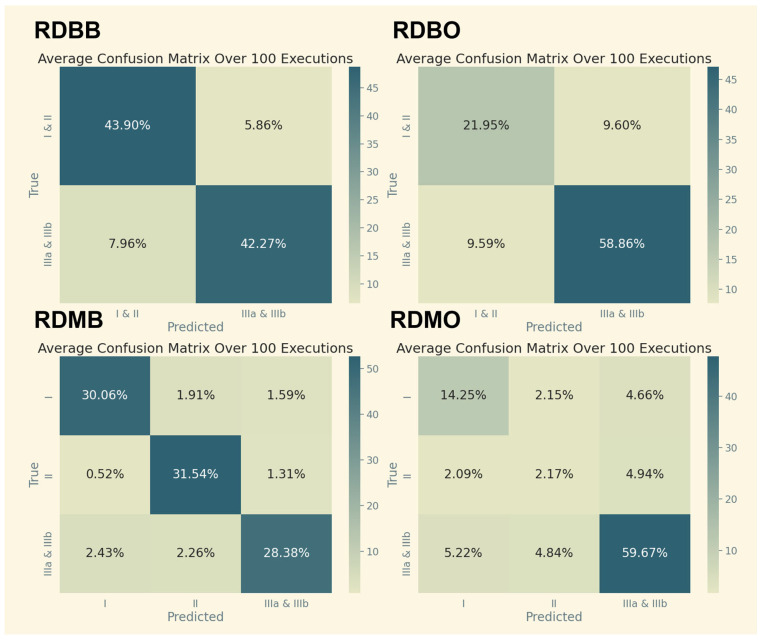
Confusion matrices from XGBoost classifications using reduced datasets averaged over 100 executions. Binary (top row) vs. multiclass (bottom row) classification; SMOTE before (left column) vs. SMOTE after (right column).

Confusion matrices for binary and multiclass classifications for different data pipelines are shown in Figure 5 and Figure 6. Multiclass classification indicated the following:

- Most confusion occurred between Stage II and Stage IIIa, likely due to biological overlap in tumor aggressiveness and nodal involvement;
- Stage I predictions were very accurate, perhaps because early-stage tumors have distinctive geometric and intensity features.

This suggests that model performance is constrained not only by data quality but by the intrinsic ambiguity of clinical staging boundaries, a known challenge even among expert radiologists.

#### 4.4.2. Error Decomposition

**Figure 7:**
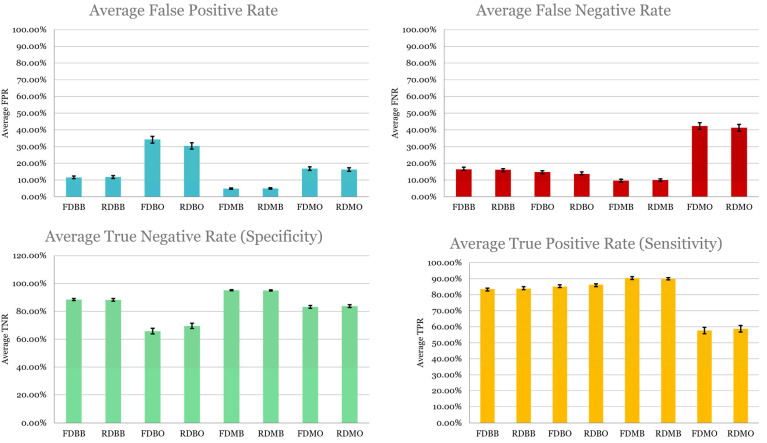
Average TPR, TNR, FNR, and FPR over 100 model executions with 95% confidence intervals.

Depicted in Figure 7, model performance was further analyzed by the following:

- True positive rate (sensitivity): The proportion of correctly identified high-stage cases;
- True negative rate (specificity): The proportion of correctly identified low-stage cases;
- False negative rate: Missed high-stage predictions;
- False positive rate: Misclassified early-stage patients.

Key insights:

- Sensitivity was highest in FDMB and RDMB, confirming their strength in detecting advanced disease, clinically critical since undertreating high-stage cancer can be fatal;
- Specificity was higher in binary models (RDBB, FDBB), possibly due to a clearer separation of low vs. high stages;
- False negative rates were highest in post-SMOTE pipelines, underscoring the risk of training on imbalanced data;
- Models using a reduced set of radiomics retained comparable sensitivity to that of the full set.

#### 4.4.3. Overall Accuracy

**Figure 8:**
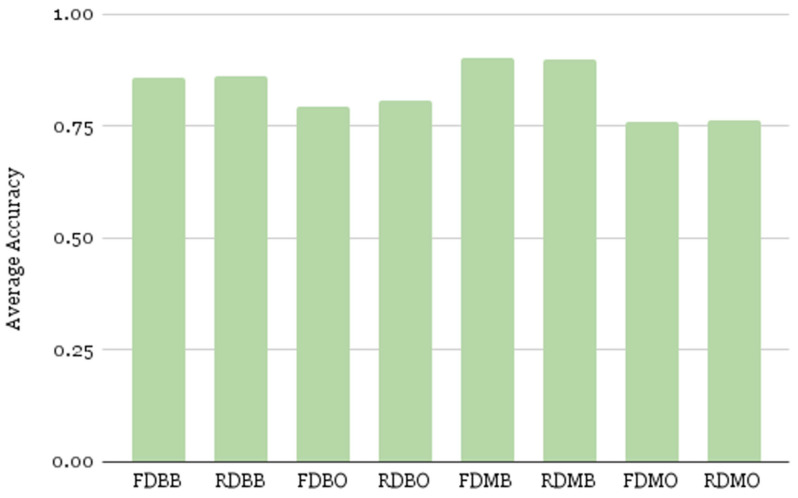
Average classification accuracy computed for 100 executions.

Depicted in Table 3 and Figure 8, SMOTE-before pipelines (B) consistently outperformed SMOTE-after (O) pipelines across both binary and multiclass settings. The RDMB pipeline achieved nearly the same accuracy as FDMB, confirming that feature reduction did not compromise performance. Binary models performed slightly lower than multiclass counterparts, likely because the three-class models leveraged finer distinctions in feature space. The poorest performance was seen in FDMO and RDMO, suggesting that delayed SMOTE reduces model learning capacity due to underrepresentation of minority classes in the training data. As shown in Table 3, the multiclass classification is performing better than the binary classification for the SMOTE-before strategy. With three distinct cancer stages in the balanced dataset, multiclass classification is more effective because synthetic data are also represented in the testing set using the SMOTE-before strategy. Particularly, as there are similar features among the cancer stages with possible overlapping distributions, data points from different cancer stages cannot be well separated into two classes and a multiclass model can better learn characteristics that distinguish between them with more complex decision boundaries.

### 4.5. Summary

Our results support the following methodological conclusions:

- A **small set of “shape” and “first-order” radiomics** can yield high diagnostic performance;
- **SMOTE-before-split** is superior for balanced learning but may risk data leakage if not carefully managed;
- **Multiclass classification** adds complexity but improves sensitivity;
- **XGBoost offers transparent, high-performing modeling** well suited for radiomics-based clinical tools.

This forms a validated technical foundation for integrating machine learning into non-invasive, imaging-based cancer staging systems.

## 5. Discussion

This study presents a rigorously constructed and experimentally validated machine learning framework for lung cancer staging using CT-derived radiomic biosensors. Our findings, obtained through systematic variation of classification strategies, oversampling procedures, and feature dimensionality, yield several important methodological insights. These insights inform not only the design of predictive models in lung cancer but also the broader use of radiomics in clinical machine learning pipelines.

### 5.1. Efficacy of Feature Selection and Reduction

One of the most practical outcomes of this study is the demonstration that only 12 radiomic features, selected through data-driven importance ranking, were sufficient to achieve classification performance comparable to the full 107-feature set. The RDMB pipeline (reduced, multiclass, SMOTE-before) achieved an average accuracy of 89.98%, just 0.32% lower than the full-feature FDMB pipeline. This remarkable reduction in dimensionality offers multiple advantages:

- Reduced computational complexity: Faster training and inference, especially beneficial in clinical settings with constrained resources;
- Improved interpretability: Clinicians can more easily trace model decisions to specific, biologically relevant features;
- Lower overfitting risk: A compact model generalizes better, especially in modest sample sizes typical of medical datasets.

For comparison with the results of the proposed pipelines, XGBoost was used to perform binary and multiclass classifications using only age, sex, and the number of tumors as predictors (reference set), along with the SMOTE-after-split oversampling strategy. The average binary classification accuracy over 100 executions was 72.79%, which was 8.02% lower than the accuracy achieved using the reduced radiomic set pipeline of the same binning and oversampling strategy (RDBO). The multiclass version of the reference set reached only 61.00% over 100 executions, which was 15.10% lower than the accuracy that was achieved using the reduced radiomic counterpart (RDMO). These figures affirm that radiomic features enhanced the predictive power of the proposed MCGBT for lung cancer stratification, especially in the case of the multiclass staging task. Moreover, because the top radiomic features are diverse (including geometric, statistical, and texture descriptors), the reduced set retains multimodal signal capacity. This supports a general design principle in radiomic modeling that cautiously selected interpretable features are often more effective than large, indistinguishable sets.

### 5.2. Impact of Class Structure and Oversampling Strategy

Another key methodological finding concerns the influence of class binning and data resampling on classifier performance. Our study compared binary and multiclass classification tasks using two SMOTE strategies, oversampling before (Strategy B) and after (Strategy O) the train–test split.

#### 5.2.1. Multiclass Classification Enhances Sensitivity

Multiclass pipelines, particularly FDMB and RDMB, consistently outperformed their binary counterparts. This indicates that more granular staging information improves model learning, perhaps by allowing better exploitation of the underlying structure in radiomic space. While multiclass problems are typically harder from a machine learning perspective, the inclusion of an intermediate stage (Stage II) may serve as a transitional buffer that helps define decision boundaries between early and late stages.

#### 5.2.2. Order of Balancing Data Procedure (SMOTE) Affects Generalization

Pipelines using SMOTE before splitting (Strategy B) outperformed those using SMOTE only on the training set. Pre-split SMOTE ensures complete class balance, which improves learning of minority class representations. However, it carries a subtle risk: data leakage may occur if synthetic examples closely resemble both training and testing instances, leading to overly optimistic performance estimates. In contrast, post-split SMOTE (Strategy O) avoids leakage but results in lower sensitivity, especially for advanced-stage tumors. This is problematic in clinical contexts where false negatives (under-staging) can lead to undertreatment. Together, these findings suggest the following:

- Pre-split SMOTE may be preferred in exploratory settings focused on identifying informative features and best-case performance;
- Post-split SMOTE may be more appropriate for validating generalization and real-world applicability.

### 5.3. Advantages of XGBoost in Clinical Modeling

Our methodological choice of XGBoost provided several clear advantages:

- Interpretable decision trees: As shown in Figure 4, XGBoost trees allowed transparent inspection of classification logic;
- Native handling of missing data and heterogeneity: The model’s robustness is well suited for radiomics, which often contains irregular feature patterns;
- Integrated feature selection: Feature importance scores were easily extracted and validated, facilitating the reduced model.

Compared to neural networks, which require large datasets and yield opaque decisions, XGBoost strikes a balance between performance and transparency, making it better aligned with the requirements of explainable AI (XAI) in healthcare.

### 5.4. Limitations and Future Directions

Despite the strengths of this methodology, its limitations must be acknowledged to be addressed in the future.

- Sample size: While relatively large with 398 records, the dataset remains modest in the machine learning domains considering the large number of predictors (including 107 radiomic features). Larger, multi-center datasets would help validate the generalizability of the identified features and the model architecture;
- Ground truth labeling: Staging labels are derived from clinical records and subject to inter-observer variability. Automated image-based staging is only as accurate as its reference labels;
- Feature standardization: Radiomic feature extraction depends on imaging parameters (e.g., voxel size, reconstruction kernel). Though PyRadiomics ensures some reproducibility [25], cross-platform consistency remains a challenge;
- Limited scope: The scope of this study was confined to a preliminary examination of eight data pipelines in conjunction with the standard XGBoost model.

Future work will address these limitations by the following:

- Incorporating multi-modal data, including PET scans and genomic markers;
- Exploring longitudinal features to predict stage progression over time;
- Evaluating generalization across external validation sets. The future work is focused on further generalization of the proposed method using other lung cancer datasets. Collaboration with cancer centers will be established to externally validate the proposed model utilizing their lung cancer data for further validation and potential customization;
- Hyperparameter tuning of the proposed MCGBT model to improve performance.

Additionally, our companion paper (in preparation) will demonstrate the clinical application of this methodology, highlighting patient-level predictions, integration into radiology workflows, and potential deployment as a decision-support tool.

## 6. Conclusions

In this study, we developed and validated a comprehensive and reproducible methodology for lung cancer staging using radiomics and XGBoost classification. By treating radiomic features as digital biosensors embedded in standard CT images, we established a framework that leverages quantitative imaging to infer tumor stage, one of the most critical determinants in cancer care.

Our pipeline systematically explored the effects of the following:

- Classification granularity (binary vs. multiclass);
- Feature dimensionality (full- vs. reduced-feature sets);
- Oversampling strategy (SMOTE before vs. after train–test split).

Through this factorial design and over 800 model executions (100 runs × 8 pipelines), we obtained a robust and statistically sound characterization of model performance under various methodological choices. The best-performing configuration, RDMB (reduced features, multiclass, SMOTE before), achieved an average accuracy of 89.98%, nearly matching the full-feature pipeline at 90.3%, with significantly reduced computational complexity and improved interpretability. It must be pointed out that the SMOTE-before strategy can lead to the creation of mislabeled synthetic patient records. This issue presents a challenge when utilizing SMOTE to create synthetic patient data for the test set, as the generated examples may not accurately reflect the true labels. However, this problem can be addressed through two main approaches, either by removing the synthetic records from the test set prior to model evaluation or performing a post-analysis of the predicted labels of the synthetic records to identify and correct any mislabeled examples.

Key methodological contributions include the following:

- Identifying shape-based radiomic features (sphericity, flatness, elongation) as predictive factors for lung cancer stage;
- Extracting a reduced set of 12 radiomics with comparable cancer staging accuracy as that of the full set of radiomics, enabling lean and deployable classifiers;
- Establishing that the order of a data-balancing procedure must be carefully contemplated. Pre-split oversampling improves the learning process, but it requires caution to avoid data leakage;
- Introducing Monte Carlo XGBoost as an efficient, interpretable, and scalable classifier with applications to medical data.

More broadly, this work advances the field of radiomics-informed machine learning in oncology. It provides a replicable template for building and evaluating intelligent imaging systems that align with the principles of precision medicine: non-invasiveness, objectivity, and clinical relevance. Our companion manuscript focuses on applying this methodological pipeline to clinical case studies and population-level analyses. Together, these two works lay the groundwork for scalable, intelligent, and transparent imaging-based cancer staging in real-world oncology practice.

## Figures and Tables

**Table 1:** Data classification pipelines.

Pipeline	Feature Set	Class Type	SMOTE Technique
FDBB	Full	Binary	Before split
FDBO	Full	Binary	After split
RDBB	Reduced	Binary	Before split
RDBO	Reduced	Binary	After split
FDMB	Full	Multiclass	Before split
FDMO	Full	Multiclass	After split
RDMB	Reduced	Multiclass	Before split
RDMO	Reduced	Multiclass	After split

**Table 2:** List of 18 radiomics in the union of four reduced-feature sets; each contain the top 12 radiomics ranked by XGBoost importance score.

Radiomic Type	Identified Features
Shape	Sphericity, elongation, flatness, maximum 3D diameter, maximum 2D diameter column, maximum 2D diameter slice
First-order stats	Maximum, minimum, median, 90th percentile
GLDM/GLCM	Dependence variance, zone entropy, energy, maximal correlation coefficient (MCC), gray-level variance 2, large-dependence low gray-level emphasis (LDLGLE)
GLRLM	Long-run low gray-level emphasis (LRLGLE)
NGTDM	Strength

Shape features dominated the top ranks, suggesting that tumor geometry is a strong indicator of stage. For example, sphericity (a measure of roundness) consistently appeared in the top 3 features across all binary and multiclass models. This aligns with biological intuition; higher-stage tumors often exhibit irregular, lobulated shapes due to invasive growth [9].

**Table 3:** Accuracy of XGBoost classification by pipeline.

Pipeline	Feature Set	Class Type	SMOTE Strategy	Accuracy (%)
FDMB	Full	Multiclass	Before Split	**90.3**
RDMB	Reduced	Multiclass	Before Split	89.98
RDBB	Reduced	Binary	Before Split	86.17
FDBB	Full	Binary	Before Split	85.85
RDBO	Reduced	Binary	After Split	80.81
FDBO	Full	Binary	After Split	79.26
RDMO	Reduced	Multiclass	After Split	76.1
FDMO	Full	Multiclass	After Split	75.96

## Data Availability

Data are publicly available and are cited in the manuscript.

## Appendix A

### Appendix A.1. Implementation of XGBoost and Hyperparameters Setup

Python implementation of XGBoost (xgboost package version 3) was performed, which supports GPU acceleration, early stopping, parallelization, and cross-validation. Model training was conducted once on 107-dimensional full-feature sets and separately on the reduced set of 12 radiomic features.

Key hyperparameters were empirically selected based on prior literature and exploratory tuning:

- objective: “binary:logistic” for binary classification; “multi:softmax” for multiclass;
- eval_metric: “logloss” for probability calibration and “mlogloss” for multiclass;
- max_depth: 6 (to balance expressiveness and overfitting);
- eta (learning rate): 0.1 (to control step size during updates);
- subsample: 0.8 (randomly sample 80% of rows per tree);
- colsample_bytree: 0.8 (randomly sample 80% of features per tree);
- lambda: 1 (L2 regularization);
- alpha: 0 (no L1 regularization);
- n_estimators: 100 (maximum number of boosting iterations).

To ensure model stability and minimize variance across random splits, each configuration (binary/multiclass, full/reduced, SMOTE strategy) was **executed 100 times** with random seeds varied in each run. Performance metrics including accuracy, sensitivity, and specificity were averaged **across multiple runs** for reliability.

### Appendix A.2. Complete List of Radiomics by Category

**Table A1:** The full list of 107 radiomics, organized by category.

**First-order statistics**	Energy
	Total energy
	Entropy
	Minimum
	10th percentile
	90th percentile
	Maximum
	Mean
	Median
	Interquartile range
	Range
	Mean absolute deviation
	Robust mean absolute deviation
	Root mean squared
	Skewness
	Kurtosis
	Variance
	Uniformity
**Shape features**	Mesh volume
	Voxel volume
	Surface area
	Surface-area-to-volume ratio
	Sphericity
	Maximum 3D diameter
	Maximum 2D diameter slice
	Maximum 2D diameter column
	Maximum 2D diameter row
	Major axis length
	Minor axis length
	Least axis length
	Elongation
	Flatness
**Gray-level co-occurrence matrix (GLCM) features**	Autocorrelation
	Joint average
	Cluster prominence
	Cluster shade
	Cluster tendency
	Contrast
	Correlation
	Difference average
	Difference entropy
	Difference variance
	Joint energy
	Joint entropy
	Informational measure of correlation (IMC) 1
	Informational measure of correlation (IMC) 2
	Inverse difference moment (IDM)
	Maximal correlation coefficient (MCC)
	Inverse difference moment normalized (IDMN)
	Inverse difference (ID)
	Inverse difference normalized (IDN)
	Inverse variance
	Maximum probability
	Sum average
	Sum entropy
	Sum of squares
**Gray-level size zone matrix (GLSZM) features**	Small area emphasis (SAE)
	Large area emphasis (LAE)
	Gray-level non-uniformity (GLN)
	Gray-level non-uniformity normalized (GLNN)
	Size zone non-uniformity (SZN)
	Size zone non-uniformity normalized (SZNN)
	Zone percentage
	Gray-level variance (GLV)
	Zone variance (ZV)
	Zone entropy (ZE)
	Low gray-level zone emphasis (LGLZE)
	High gray-level zone emphasis (HGLZE)
	Small area low gray-level emphasis (SALGLE)
	Small area high gray-level emphasis (SAHGLE)
	Large area low gray-level emphasis (LALGLE)
	Large area high gray-level emphasis (LAHGLE)
**Gray-level run length matrix (GLRLM) features**	Short-run emphasis (SRE)
	Long-run emphasis (LRE)
	Gray-level non-uniformity 1 (GLN1)
	Gray-level non-uniformity normalized 1 (GLNN1)
	Run length non-uniformity (RLN)
	Run length non-uniformity normalized (RLNN)
	Run percentage (RP)
	Gray-level variance 1 (GLV1)
	Run variance (RV)
	Run entropy (RE)
	Low gray-level run emphasis (LGLRE)
	High gray-level run emphasis (HGLRE)
	Short-run low gray-level emphasis (SRLGLE)
	Short-run high gray-level emphasis (SRHGLE)
**Neighboring gray tone difference matrix (NGTDM) features**	Long-run low gray-level emphasis (LRLGLE)
	Long-run high gray-level emphasis (LRHGLE)
	Coarseness
	Contrast 1
	Busyness
**Gray-level dependence matrix (GLDM) features**	Complexity
	Strength
	Small-dependence emphasis (SDE)
	Large-dependence emphasis (LDE)
	Gray-level non-uniformity 2 (GLN2)
	Dependence non-uniformity (DN)
	Dependence non-uniformity normalized (DNN)
	Gray-level variance 2 (GLV2)
	Dependence variance (DV)
	Dependence entropy (DE)
	Low gray-level emphasis (LGLE)
	High gray-level emphasis (HGLE)
	Small dependence low gray-level emphasis (SDLGLE)
	Small-dependence high gray-level emphasis (SDHGLE)
	Large-dependence low gray-level emphasis (LDLGLE)
	Large-dependence high gray-level emphasis (LDHGLE)
